# Effect of waterpipe tobacco smoke exposure on the development of metabolic syndrome in adult male rats

**DOI:** 10.1371/journal.pone.0234516

**Published:** 2020-06-19

**Authors:** Nour A. Al-Sawalha, Yehya Almahmmod, Mofleh S. Awawdeh, Karem H. Alzoubi, Omar F. Khabour

**Affiliations:** 1 Department of Clinical Pharmacy, Faculty of Pharmacy, Jordan University of Science and Technology, Irbid, Jordan; 2 Department of Veterinary Pathology & Public Health, Faculty of Veterinary Medicine, Jordan University of Science and Technology, Irbid, Jordan; 3 Department of Medical Laboratory Sciences, Faculty of Applied Medical Sciences, Jordan University of Science and Technology, Irbid, Jordan; West Virginia University, UNITED STATES

## Abstract

The prevalence of metabolic syndrome is increased worldwide. Tobacco smoking increases the risk of developing metabolic syndrome. Waterpipe tobacco smoking has become a global trend of tobacco consumption and is as common as cigarette smoking. In this study, the effect of waterpipe tobacco smoke (WTS) on the development of metabolic syndrome in rats was evaluated. Adult Wistar rats were exposed for 19 weeks to either fresh air (control) or WTS for 1 hour daily/ 5 days per week (WTS). Central obesity, systolic blood pressure, lipid profile, glucose hemostasis and levels of leptin and adiponectin were evaluated. The WTS exposure increased body weight, abdominal circumference, systolic blood pressure and fasting glucose compared to control animals (P<0.05), consistent with inducing metabolic syndrome. The retroperitoneal fat, lipid profile and levels of insulin, leptin and adiponectin were not affected by WTS exposure (P>0.05). In conclusion, exposure to WTS has detrimental health effects leading to the development of metabolic syndrome in experimental animals.

## Introduction

Metabolic syndrome is a group of health problems that are caused by genetic and environmental factors [1]. A pooled analysis revealed that about 4.8–7% of young adults have metabolic syndrome [2]. The prevalence of metabolic syndrome was about 25% in Middle-East countries based on a recent meta-analysis [3]. The observed variation in the prevalence of metabolic syndrome could be due to the studied population, the used definition of metabolic syndrome and the presence of environmental risk factors such as physical activity and smoking [4]. The diagnosis of metabolic syndrome varies according to the used definition. There are several criteria that are used to diagnose metabolic syndrome, but in general, the presence of 3 out of the following 5 criteria qualifies the diagnosis of metabolic syndrome; hyperglycemia, elevated triglycerides level, reduced high-density lipoprotein (HDL) cholesterol, hypertension and central adiposity [5]. Metabolic syndrome predisposes the individual to develop several diseases such as cardiovascular diseases, diabetes [6], renal and hepatic complications among others [4].

Several studies showed that cigarette smoking is associated with metabolic syndrome [7, 8]. According to WHO, there are around 1.1 billion smokers worldwide who live in low- and middle-income countries [9]. In the last decade, waterpipe smoking globally has become as common as cigarette smoking. According to a recent systematic review, waterpipe tobacco smoking is most prevalent in European and Eastern Mediterranean countries, especially among the youth population [10]. Several studies showed that the prevalence of WTS is higher among males compared to females (Abu Seir et al., 2020; Akl et al., 2011; Grekin & Ayna, 2012; Salloum et al., 2019).

The waterpipe users have the misconception that waterpipe smoking is less harmful and addictive than cigarette smoking as well as considering it as an essential part of social gathering [11]. These factors contribute to the noticed increased prevalence of WTS. Waterpipe smoking is associated with bladder cancer, nasopharyngeal cancer, oral dysplasia, and infertility [12]. WTS contains a profile of toxicants that impacts body health such as nicotine, heavy metals (like arsenic, chromium and lead), tar [13] and carbon monoxide [14].

A cross-sectional study showed that waterpipe smokers were more likely to have metabolic syndrome as compared to nonsmokers after adjusting the analysis for age, sex and social class [15]. Waterpipe smokers had higher levels of triglyceride, glucose, blood pressure and abdominal obesity than non-smokers [15]. In addition, waterpipe smokers had a higher body mass index as compared to nonsmokers [16]. However, the recall bias and ignorance of the influence of lifestyle habits might affect the studied association between waterpipe smoking and the development of metabolic syndrome. Therefore, examining the effect of exclusive exposure to WTS on the development of metabolic syndrome utilizing an animal model was the goal of this study.

## Methods

### Animals

Young adult male Wistar rats, 200–250g, were purchased from the Animal Care Unit at Jordan University of Science and Technology (JUST) (Irbid, Jordan). All experimental procedures were approved by the Animal Care and Use Committee (ACUC) of Jordan University of Science and Technology. Rats were kept in a 12:12 light/dark cycle at standard room temperature with free access to water and food. The rats were acclimatized for 10 days before starting the experiment. Rats (n = 20) were randomly assigned to either receive fresh air (control) or WTS for 19 weeks.

### WTS exposure

Animals in waterpipe groups were exposed to mainstream waterpipe for 1 hour twice daily for 5 days/week for 19 weeks using whole-body exposure system as described previously [17]. Briefly, the apparatus is composed of exposure chamber, diaphragm pump and WTS apparatus. The diaphragm draws the smoke into the exposure chamber. Around 10 grams of Two Apples^®^ flavor tobacco (Nakhla brand, Egypt) was used in the waterpipe head during each WTS session. Quick-light charcoal briquettes were the used heat source. Total particulate matter in the mainstream smoke was measured using a 47 mm glass fiber filter (Pall Type A/E) as previously described [18]. The mean total particulate matter per 1 hour session was 1319 ± 361 mg/m^3^. In each exposure session, carbon monoxide (CO) concentration was adjusted to maintain the exposure to all animals at similar concentrations (1033 ± 87 ppm, mean ± SD).

### Body weight and central obesity measurements

Weight and abdominal circumference were measured at week 19. The weight of the excised retroperitoneal white adipose tissues were used to assess the central obesity as described previously [19]. The abdominal circumference in animals, that represents waist circumference in human, is also an indicator of abdominal obesity [20].

### Systolic blood pressure measurement

Systolic blood pressure was measured at week 19 utilizing the tail-cuff plethysmography method (Computerized tail-cuff plethysmography blood pressure system, IITC Life Science, Woodland Hills, CA). The animals were acclimatized for 10 days before measuring systolic blood pressure. A special software (IITC software version 1.1, IITC Life Science) was used to record the systolic blood pressure.

### Lipid profile, glucose hemostasis and hormonal levels

Rats were euthanized by rapid decapitation without anesthesia since anethsia affect the lipid profile [21]. The serum was separated and stored at -80°C until further analysis. Serum level of triglyceride (Biosystems, Barcelona, Spain), total cholesterol (TC) (Biosystems, Barcelona, Spain), and HDL cholesterol (Biosystems, Barcelona, Spain) were measured using the available commercial kits according to the manufacturer instruction. Friedewald’s low-density lipoprotein (LDL)-cholesterol estimation formula [22] was utilized to calculate the level of LDL.

Fasting blood glucose was measured from rat-tail by blood glucose meter (ACCU-CHEK, Roche diagnostic, USA). Insulin concentration was determined by the commercially available ELISA kit (MyBioSource, SanDiego, CA, USA) according to the manufacturer’s instructions. Serum levels of leptin (R&D Systems, USA) and adiponectin (R&D Systems, USA) were measured by ELISA kits following the manufacturers’ instructions. The absorbance was read at the appropriate wavelength by Epoch Biotek microplate reader (BioTek, Winooski, VT, USA).

### Statistics

Data were presented as mean ± standard error of the mean (SEM). The data were checked for normality by D’Agostino & Pearson omnibus and Shapiro-Wilk normality tests. Student’s t-test or Mann-Whitney tests were used for the comparison between the two groups. P≤0.05 was considered statistically significant. The statistical analysis was performed utilizing GraphPad Prism 5^®^ software.

## Results

### Effect of WTS exposure on body weight, central obesity and systolic blood pressure

There was no statistical difference in the baseline body weight and abdominal circumference among control and WTS groups (P>0.05). WTS exposure for 19 weeks significantly increased the abdominal circumference (P<0.0001) and body weight (P = 0.02) compared to control group. However, there was a trend of reduced the retroperitoneal fat in animals that were exposed to WTS, but it did not reach a significant level, compared to control (P = 0.06). Table 1 summarizes the effect of WTS exposure on body weight, abdominal circumference and retroperitoneal fat.

**Table 1:** Effect of WTS on body weight, abdominal circumference and retroperitoneal fat.

	Pre-exposure		Post-exposure		% Change	
Parameter	Control	WTS	Control	WTS	Control	WTS
**Body weight (g)**	159.9±1.8	160.4±0.32	345.6±11.0	378.6±11.9*	116.5±8.1%	136.1±7.5%*
**Abdominal circumference (cm)**	12.4± 0.06	12.2±0.05	18.5±0.2	20.4±0.2*	48.9± 1.8%	66.5±1.2%*
**Retroperitoneal fat (g)**	-	-	3.7±0.3	2.9±0.2	-	-

* Indicates significant difference from control group. Values were expressed as mean ± SEM from 8–10 animals. *P* < 0.05 was considered statistically significant.

**Fig 1:**
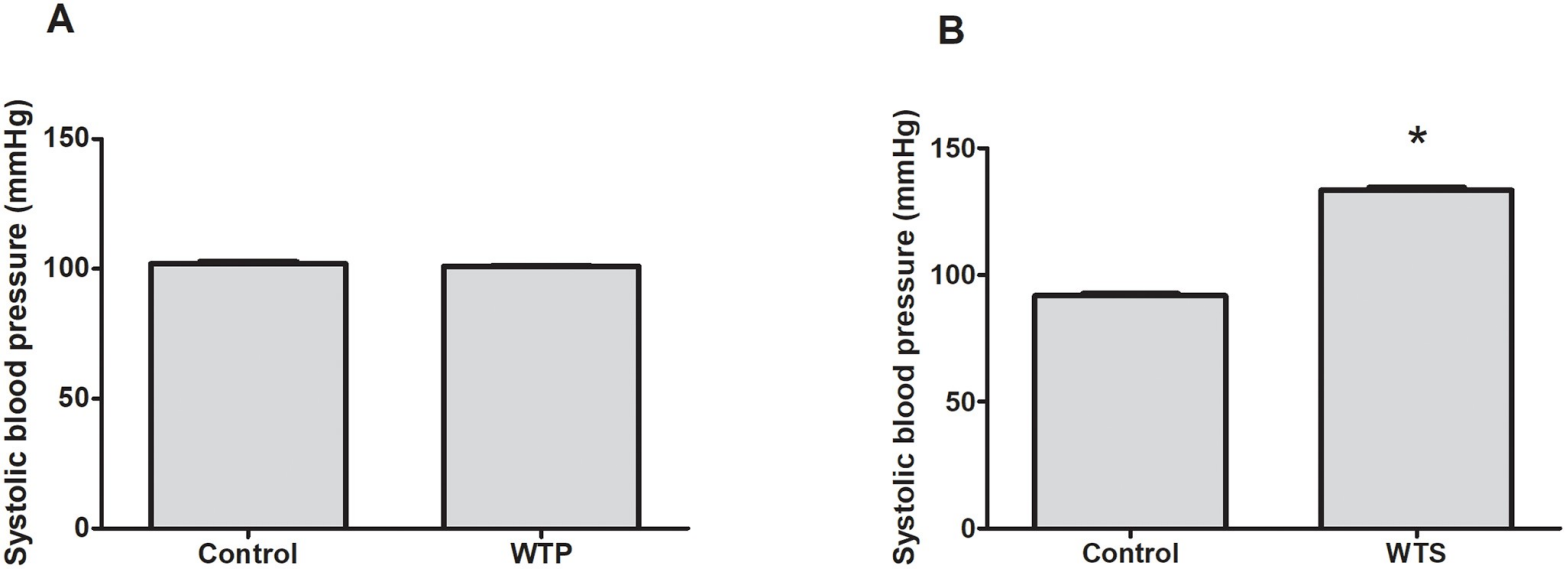
Effect of WTS on systolic blood pressure. Systolic blood pressure was measured at (A) baseline and (B) after exposure to fresh air or WTS for 19 weeks * indicates significant difference from control. Values are expressed as mean ± SEM of 8 animals per group. P < 0.05 was considered statistically significant.

Further, the baseline systolic blood pressure among the control and WTS groups was not statistically significant (102.0±0.9 mmHg in control group versus 101.0±0.4 mmHg in WTS group p>0.05) (Fig 1A). Exposure to WTS increased systolic blood pressure significantly compared to control (91.9±0.7 mmHg in control group versus 133.4±1.2 mmHg in WTS group, P<0.0001) (Fig 1B).

### Effect of WTS exposure on lipid profile

Exposure to WTS did not affect the level of triglycerides, total cholesterol, LDL and HDL compared to control group (Table 2).

**Table 2:** Effect of WTS on lipid profile, glucose homeostasis and hormonal levels.

Parameter	Control	WTS
**Triglycerides (mg/dl)**	33.1±3.7	53.8±14.9
**Total Cholesterol (mg/dl)**	70.7±4.1	76.9±5.0
**LDL (mg/dl)**	9.5±1.8	11.3±4.0
**HDL (mg/dl)**	54.6±3.8	54.8±3.8
**Fasting glucose (mg/dl)**	63.0±1.0	91.7±1.4*
**Insulin (pg/ml)**	134.8±32.3	107.1±30.2
**Leptin (ng/ml)**	1.5±0.6	0.9±0.2
**Adiponectin (ng/ml)**	17221 ±2474	23414±2355

* Indicates significant difference from control group. Values were expressed as mean ± SEM from 8–10 animals, except insulin from 5–8 animals. *P* < 0.05 was considered statistically significant.

### Effect of WTS exposure on glucose homeostasis, leptin and adiponectin levels

Rats that were exposed to WTS showed elevated levels of fasting blood glucose compared to the control group (P<0.0001). The level of insulin was not affected by WTS exposure compared to control (P = 0.6). The exposure to WTS showed a trend of reduced leptin level and increased adiponectin level (P = 0.09) compared to control, but it did not reach a significant level. (Table 2).

## Discussion

This study showed the detrimental effect of chronic exposure to WTS on the manifestations of metabolic syndrome. The current study showed that WTS exposure increased body weight, abdominal circumference, systolic blood pressure and fasting glucose compared to control animals.

The results of the current study showed that chronic WTS exposure increased body weight and abdominal circumference in animals, conistent with human observation [23]. Central obesity can be assessed by retroperitoneal fat [19] or waist (abdominal) circumference [20]. Body weight and central obesity are controlled by several hormones and molecules including adipokines; leptin and adiponectin. Recent reports suggested that leptin [24] and adiponectin [25] play important roles in the development of the metabolic syndrome. Leptin is secreted mainly from the differentiated adipocytes [26]. Leptin suppresses the appetite and food intake by reducing the level of neuropeptide Y, increasing energy expenditure and decreasing body weight [27]. The circulatory concentration of leptin is mainly determined by the body fat mass [28]. In the current study, WTS exposure slightly reduced the concentration of leptin compared to control and this could be explained by slightly reduced retroperitoneal fat, though it did not reach a significant level. The unaltered concentration of leptin by WTS exposure was consistent with the exposure to cigarette smoke [29]. However, there are other determinants of the concertation of leptin other than body weight, such as ghrelin, resistin and glucagon like-peptide 1. Male rats were only studied in the current study as metabolic syndrome is more prevalent in males than females as estrogen provides a protective effect against the development of metabolic syndrome [30, 31]. However, future studies should examine the effect of sex on the susceptibility to develop metabolic syndrome by chronic WTS exposure.

Adiponectin, another important adipokine that is involved in regulating body weight, is secreted by adipocytes and controls body weight through decreasing lipid, glucose and triglyceride production as well as increasing fat oxidation [32, 33]. Obesity decreases the circulating level of adiponectin [34]. The current study showed a trend of increased adiponectin concentration by WTS exposure, although it was not significant. A systematic review of adult human studies revealed that cigarette smoking decreased the concentration of adiponectin [35]. The inconsistency of the results could be due to the difference between cigarette smoking and WTS and human versus animal models.

Several studies revealed that waterpipe smoking increased the blood pressure, heart rate and eventually the risk of overall cardiovascular events [36, 37]. The current study showed increased systolic blood pressure in animals that were exposed to chronic WTS, consistent with Nemmar and colleagues finding [38]. However, future studies should examine the hormonal changes as well as the molecular targets that underlines the increased systolic blood pressure as L-type calcium channel kinetics in vascular smooth muscle cells and renin-angiotensin-aldosterone system.

Waterpipe smoking altered lipid profile, where it increased the levels of triglycerides and LDL [39]. However, the current study revealed that WTS exposure increased the level of triglycerides, total cholesterol and LDL, but did not reach a significant level. This inconsistency in results could be due to the differences between human data and animal models.

It has been shown that WTS is associated with the development of diabetes [40]. The current study revealed that WTS increased the level of fasting blood glucose consistent with previous studies [15, 41]. However, the serum level of insulin was not affected. Glucose level is not only affected by insulin, there are other hormones that influence glucose level as cortisol, glucagon and somatostatin. Further work is needed to examine a panel of different hormones that affect glucose homeostasis.

The results in the current study points toward the effect of WTS exposure on the development of the metabolic syndrome’s components, consistent with previous studies [15, 41]. The molecular mechanisms by which WTS induced metabolic syndrome phenotype was not investigated in the current study. However, it is postulated that WTS induced oxidative stress that affect the activity of transcription factors, induced the release of stress hormones and eventually contribute to the development of several diseases [42].

The current study has several limitations. The levels of nicotine, cotinine and other components of WTS were not measured in the animals at the end of the exposure period. Further, the level of glycosylated hemoglobin was not measured to give more insights into the glucose homeostasis. In addition, the oxidative stress biomarkers were not measured in the current study as there are several reports showed that oxidative stress plays an important role in the development of metabolic syndrome [43].

In conclusion, the current study showed that chronic exposure to WTS induced the manifestations of metabolic syndrome as increased abdominal circumference, systolic blood pressure and fasting glucose. The results highlight the detrimental health effect of chronic exposure to WTS.

## Supporting information

S1 Dataset Dataset is an excel file that includes the used data to generate figures and tables of the study. (XLSX)
